# Machine Learning–Based Signal Quality Evaluation of Single-Period Radial Artery Pulse Waves: Model Development and Validation

**DOI:** 10.2196/18134

**Published:** 2020-06-22

**Authors:** Xiaodong Ding, Feng Cheng, Robert Morris, Cong Chen, Yiqin Wang

**Affiliations:** 1 Shanghai Key Laboratory of Health Identification and Assessment Laboratory of Traditional Chinese Medicine Four Diagnostic Information Shanghai University of Traditional Chinese Medicine Shanghai China; 2 Department of Pharmaceutical Science College of Pharmacy University of South Florida Tampa, FL United States

**Keywords:** pulse wave, quality evaluation, single period, segmentation, machine learning

## Abstract

**Background:**

The radial artery pulse wave is a widely used physiological signal for disease diagnosis and personal health monitoring because it provides insight into the overall health of the heart and blood vessels. Periodic radial artery pulse signals are subsequently decomposed into single pulse wave periods (segments) for physiological parameter evaluations. However, abnormal periods frequently arise due to external interference, the inherent imperfections of current segmentation methods, and the quality of the pulse wave signals.

**Objective:**

The objective of this paper was to develop a machine learning model to detect abnormal pulse periods in real clinical data.

**Methods:**

Various machine learning models, such as k-nearest neighbor, logistic regression, and support vector machines, were applied to classify the normal and abnormal periods in 8561 segments extracted from the radial pulse waves of 390 outpatients. The recursive feature elimination method was used to simplify the classifier.

**Results:**

It was found that a logistic regression model with only four input features can achieve a satisfactory result. The area under the receiver operating characteristic curve from the test set was 0.9920. In addition, these classifiers can be easily interpreted.

**Conclusions:**

We expect that this model can be applied in smart sport watches and watchbands to accurately evaluate human health status.

## Introduction

Pulse-taking is widely used in disease diagnosis and personal health monitoring. For example, in traditional Chinese medicine (TCM), pulse-taking is an important approach to differentiate TCM syndrome patterns in which the physician uses their fingers to detect patients’ pulsations. The radial artery is the most frequently used position for pulse-taking because the pulse wave of the radial artery contains abundant physiological information and is convenient for pulse-taking due to the accessibility of the vessels [1]. The development of smartwatches in recent years, coupled with pulse-taking analysis applications, enables individuals to monitor their pulse rates and physiological state throughout the day. As the number of smartwatch users expands, researchers are increasingly attempting to detect a variety of subclinical diseases such as atrial fibrillation (AF) by radial artery pulse waves in the early stages of disease progression. However, the majority of existing approaches are based on heart rate variability, which ignores important information contained in the changing pulse wave during a single cardiac cycle. The deep neural network models used for prediction are sometimes difficult to interpret [2]. Recently, researchers in the field of TCM diagnostics and hemodynamics have successfully utilized the information contained in single-period pulse waves not only to differentiate traditional syndrome patterns and diseases such as hypertension, diabetes, and other diseases not directly related to heart rhythm but also to fit modern clinical indices through objective recording [3-7]. Incorporating single-period pulse wave signals in smartwatches may improve the accuracy of existing applications in an interpretable way and expand the application scope of radial artery pulse waves. 

**Figure 1:**
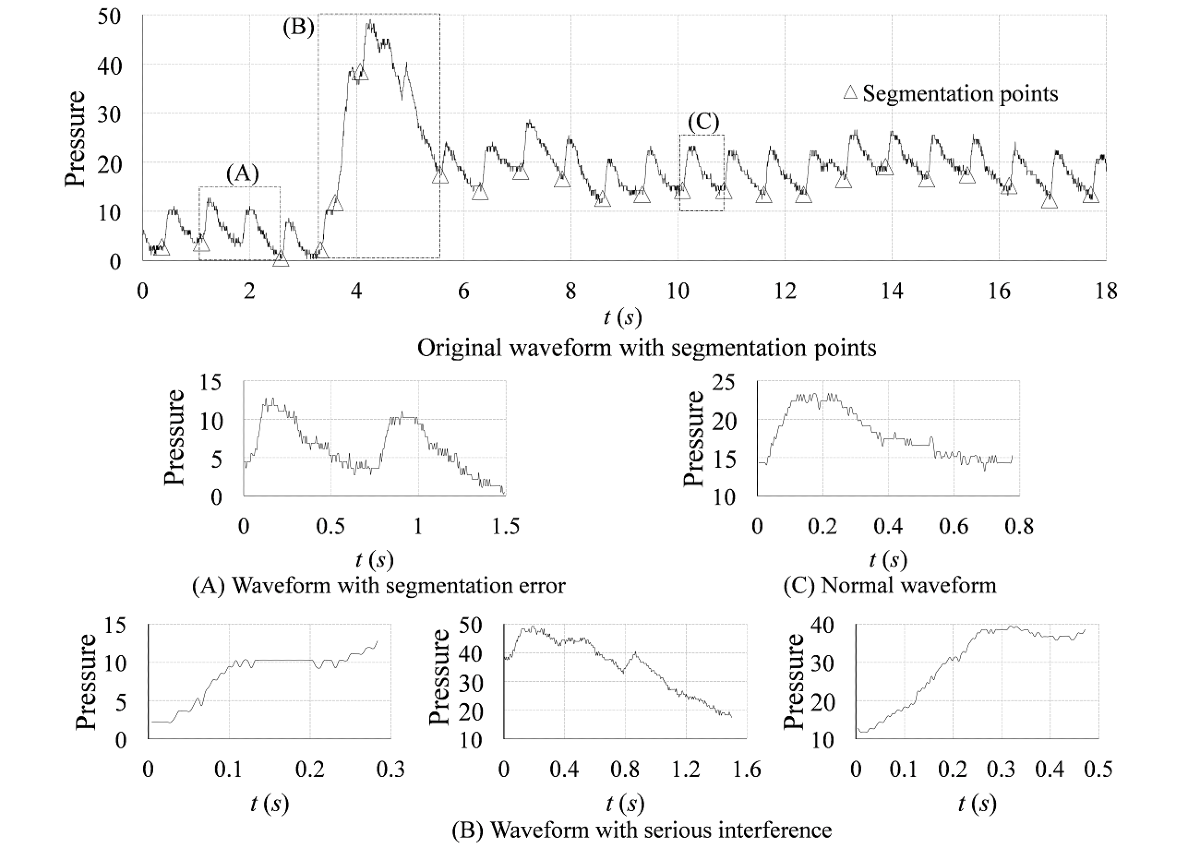
Normal and abnormal pulse wave segments. A radial artery pulse wave series was segmented into periods by the segmentation method detailed in the Preprocessing section with α=.7. The segments of the original waveform between two adjacent segmentation points are regarded as single-period pulse waveforms. A and B show the abnormal segments caused by segmentation error and serious interference, respectively; C shows a normal segment; *t* (*s*): time in seconds.

In general, the radial artery pulse wave is a periodic signal. Therefore, it is necessary to segment the whole pulse wave series into periods before performing data mining of single-period pulse waves. However, the radial artery pulse wave signal is so weak that it is vulnerable to interference (such as breathing or vibration) during the acquisition process. These interferences can lead to waveform distortion, which increases the difficulty of segmenting the periods. In addition, no currently existing algorithm can extract single-period pulse wave signals from whole pulse wave series without error. Therefore, some pulse wave segments (abnormal segments) obtained by period segmentation are often remarkably different from the normal waveforms (Figure 1). These abnormal waveform pulses may affect future prediction results. As a result, automatically identifying these outliers from single-period pulse wave signals will significantly improve the accuracy of analysis.

The early approach was to omit the waveform outliers that were too long or too short [8]. However, this method cannot be used to identify outliers with normal lengths. Thakker and Vyas [9] used dynamic time warping as a similarity measure in a pulse series in which the most dissimilar segments in the pulse series were classified as outliers. However, for patients with atrial fibrillation or other specific diseases, abnormal waveforms often appear in one series. In addition, significant outside interference may drastically reduce the number of normal segments. Due to these factors, it is difficult to discriminate between normal signals and outliers using this algorithm. This similarity is not the only important criterion for classifying pulse segments. Wang and Lu [10] utilized a k-nearest neighbor (KNN) classifier based on manual label data to measure the quality of the segmented single periods. However, the details and accuracy of the classifier were not shown. In a recent study, a method based on the Hilbert-Huang transform and an autoregressive moving average model was proposed to remove noise-induced mutations [11]. The accuracy of this method could reach 91.8% in a sample size of 207. However, because this method works on the entire pulse series, segmentation mistakes could not be identified. A consensus on the best method to evaluate the signal quality of a single-period pulse wave has not been reached. The purpose of this study was to utilize machine learning models to develop a signal quality evaluation model that can separate normal segments and abnormal segments. We expect that the model can be applied to help smart sport watches and watchbands evaluate human health status more accurately.

## Methods

### Data

In this study, the original radial artery pulse wave signals were taken from 390 outpatients at Shanghai Shuguang Hospital. All samples were split into an 80/20 ratio for training and testing sets. In other words, the data set was randomly divided into a training set with a capacity of 313 and a testing set with a capacity of 77.

### Preprocessing

**Figure 2:**
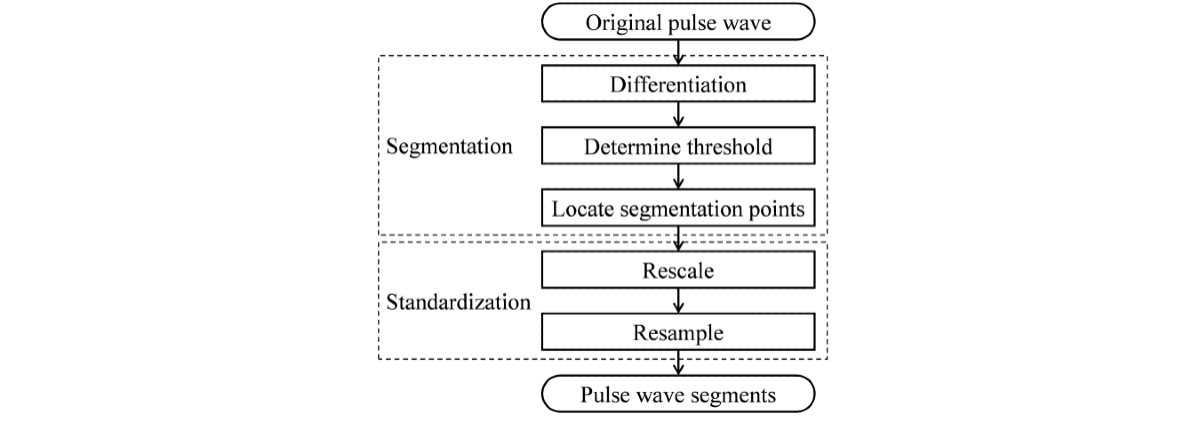
The steps of data preprocessing. During segmentation, to reduce the influence of baseline wander, the derivative of the original signal was used to locate segmentation points by the threshold method. The corresponding segments of the original signal between two adjacent period segmentation points were single-period pulse wave segments. During standardization, each segment was rescaled and resampled to standardize its amplitude and length.

The steps of preprocessing, including segmentation and standardization, are illustrated in Figure 2.

**Figure 3:**
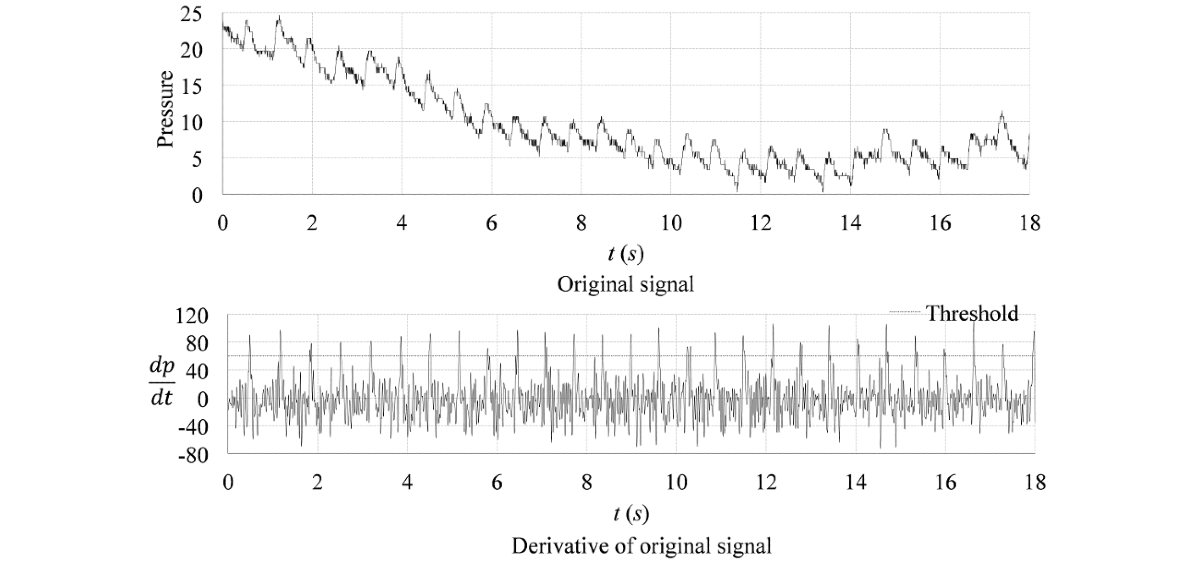
Sample pulse wave with baseline wander and the derivative of the pulse wave with an applied threshold. *t* (*s*): time in seconds.

A simple segmentation method is the threshold method, which regards the minimum point below a specific threshold or the maximum point above a specific threshold as the period segmentation point. However, baseline wander is one of the most common forms of interference in pulse wave signals. Thus, it is difficult to apply the threshold method to the original signal. In contrast, as shown in Figure 3, the derivative of the original signal is almost entirely unaffected by baseline wander and also shows clearer segmentation points. Therefore, the threshold method can be used in the derivative for segmentation.

To collect more normal and abnormal segments with different shapes, 5 different thresholds (between the maximum value of the derivative and 0) were selected for segmentation. If M is the maximum value of the derivative and α is a coefficient between 0 and 1, the threshold is given by

threshold = Mα (**1**)

In this study, for each patient’s data, α takes 5 different values: 0.1, 0.3, 0.5, 0.7, and 0.9.

During segmentation, all threshold points of the derivative were first found. The first zero point of the derivative before each threshold point was defined as the period segmentation point, and the corresponding segments of the original signal between two adjacent period segmentation points were single-period waveforms. To avoid the presence of repetitive waveforms with significant similarities in the data set, only 5 segments were randomly selected for each threshold (if sufficient). No more than 25 segments were obtained from each patient by repeating the above process with different thresholds.

After segmentation, each segment was manually labeled as “normal” or “abnormal” by two expert annotators. A normal segment was required to have similar lengths, amplitudes, and shapes to most other segments in the same pulse series; be free of serious interference that could not be explained by the laws of physiology and pathology; have an approximately horizontal baseline, in which the difference between the values of the start point and the end point was not more than half the amplitude; and include only one complete cardiac cycle. The segments in which the two experts could not reach a consensus on their labels were not included in subsequent analyses. A total of 6832 segments were collected in the training set, of which 3974 (58.2%) were normal segments and 2858 (41.8%) were abnormal segments. In addition, a total of 1729 segments were collected in the testing set; 965 normal segments (55.8%) and 764 abnormal segments (44.2%) were identified.

The amplitudes and lengths of the segments differed from one another. To reduce the impact on the classification process, the original signals were standardized before classification. The segments were rescaled so that all values fell in the interval (0,1). If *X =* {*x*_1_, *x*_2_, …, *x*_n_} was a segment, the rescaled segment *Y* was given by

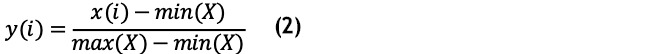



Cubic spline interpolation was used to resample the segments to unify their lengths to a standard length. In this study, the trial values for the standard length were integers between 3 and 100. In general, the frequency range that contains useful information is below 25 hertz, and the cardiac cycle is no longer than 2 seconds. 100 sampling points are sufficient to contain all the useful information in one period. Hence, integers greater than 100 were not tested in this study.

### Classification Methods

All 100 sampling points of the pulse wave segments were used as input features of the classifiers in this study. Three machine learning algorithms were applied to develop the classifiers:

1. KNN: The n_neighbors parameter was determined through cross-validation; the trial values for n_neighbors were integers in the range of 1-50.
2. Logistic regression: To reduce the influence of multicollinearity, L2 regularized logistic regression was chosen.
3. Support vector machine: support vector machine models with the radial basis function kernel (SVM-RBF), linear kernel (SVM-Linear), and 3-degree polynomial kernel (SVM-Poly) were applied. The cost and gamma parameters were determined through cross-validation, with trial cost values of 0.01, 0.1, 1, 10, and 100 and trial gamma values of 0.001, 0.01, 0.1, 1, and 10.

To estimate the accuracy of the models (out-of-sample accuracy), 10-fold cross-validation was applied. The most appropriate machine learning model was chosen by comparing the sensitivity, specificity and accuracy of the different algorithms. Based on the selected model, a reasonable value of standard input length was then identified by comparing the classification accuracies with different standard lengths.

In addition, the pulse waveforms that point at different positions in a cardiac cycle have different physiological significance and may influence the signal quality evaluation to varying degrees. To evaluate the contribution of each feature to the classification, recursive feature elimination was used to rank the features [12,13]. By excluding features one by one, it is possible to identify the smallest subset of features that can achieve satisfactory classification performance.

All the above steps were implemented in the training set. The final prediction model was validated on the testing set.

## Results

### Accuracy, Sensitivity, and Specificity of the Three Classification Models

The maximum accuracy, sensitivity, and specificity of each classification algorithm are presented in Table 1. All values are greater than 0.94, which indicates that all three algorithms performed similarly and effectively in waveform classification. To increase the simplicity and interpretability of the model, logistic regression was selected for further investigation.

**Table 1:** Comparison of the three classification algorithms, 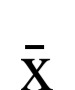
±σ.

Method		Accuracy	Sensitivity	Specificity
KNN^a^		0.9732±0.0086	0.9901±0.0085	0.9494±0.0066
Logistic regression		0.9698±0.0122	0.9801±0.0141	0.9546±0.0128
**Support vector machine**				
	SVM-RBF^b^ (cost=10, gamma=0.1)	0.9797±0.0084	0.9862±0.0127	0.9691±0.0074
	SVM-Linear^c^ (cost=0.1, gamma=0.001)	0.9703±0.0124	0.9814±0.0137	0.9540±0.0140
	SVM-Poly^d^ (cost=1, gamma=0.1)	0.9756±0.0103	0.9866±0.0110	0.9594±0.0109

^a^KNN: k-nearest neighbors; n=6.

^b^SVM-RBF: support vector machine-radial basis function kernel.

^c^SVM-Linear: support vector machine-linear kernel.

^d^SVM-Poly: support vector machine-3-degree polynomial kernel.

### Standard Length

**Figure 4:**
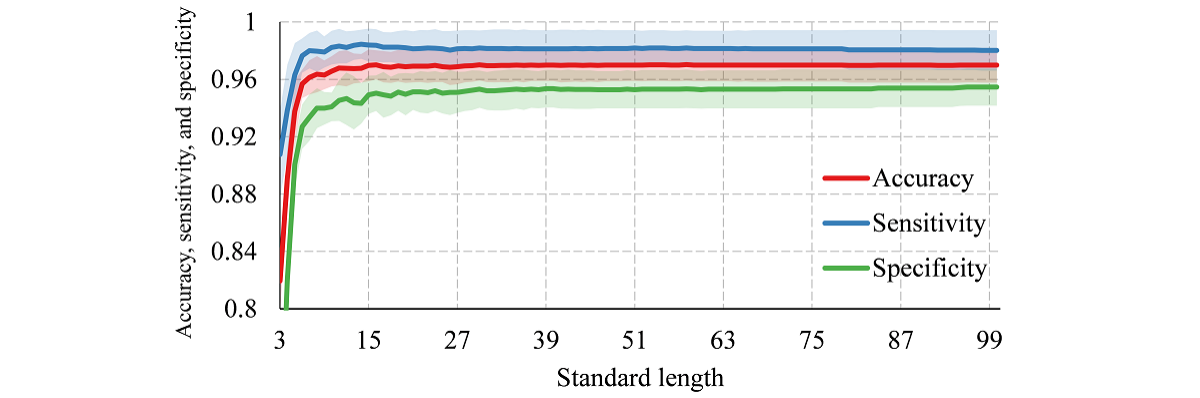
Accuracy, sensitivity, specificity, and standard deviation intervals of the classifier with different standard lengths.

Figure 4 shows the performance of the logistic regression model with different standard lengths. When the standard length was >15, the model was stable and performed well; when the standard length was <15, the performance of the classifier deteriorated gradually as the standard length decreased. Therefore, 15 was selected as a reasonable value of the standard length for simplification of the model.

**Figure 5:**
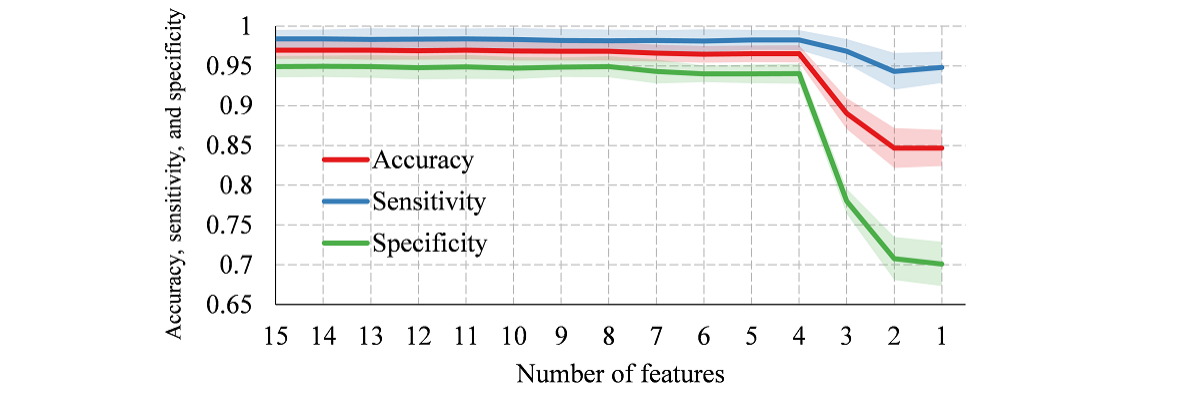
Accuracy, sensitivity, and specificity of the classifier with sequentially eliminated input features and their standard deviation intervals.

Each segment was resampled using the standard length of 15, and the importance ranking of the 15 feature points (Table 2) was then calculated based on the recursive feature elimination algorithm. When we sequentially eliminated the features one by one, as illustrated in Figure 5, the classifier performed equally well when the number of features was equal to or greater than 4. As shown in Table 2, the four most important feature points are the third, 14th, fourth, and first feature points. That is to say, a satisfactory logistic regression classification model can be established by using only four features (the third, 14th, fourth, and first points of the 15 feature points).

**Table 2:** Importance ranking of the feature points.

Importance ranking	Position of the feature point
1	3
2	14
3	4
4	1
5	7
6	15
7	13
8	11
9	6
10	9
11	10
12	2
13	8
14	5
15	12

### Final Prediction Model

If *x_n_* is the value of the nth feature point, and *P* is the probability that the segment is normal, the logistic regression classifier identified based on the training set can be given by

*P* = *sigmoid*(–9.6919*x*_1_ + 8.2570*x*_3_ + 8.9216*x*_4_ – 7.9818*x*_14_ – 10.8732) (**3**)

where

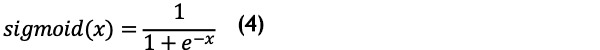



The prediction model indicates that when *x*_1_ and *x*_14_ are close to 0 and *x*_3_ and *x*_4_ are sufficiently large, the corresponding pulse wave segment can be classified as normal.

**Figure 6:**
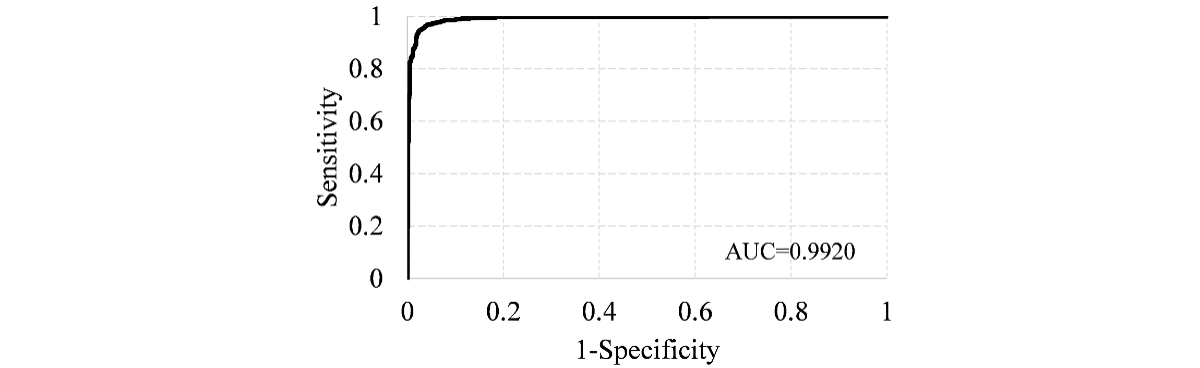
ROC curve of the final logistic regression classifier on the testing set. AUC: area under the curve.

We applied this classifier to the testing set, and the receiver operating characteristic (ROC) curve is shown in Figure 6. The area under the curve (AUC) was 0.9920. Using the default threshold of 0.5, the accuracy, sensitivity, and specificity of the classifier were 0.9607, 0.9741, and 0.9437, respectively. This is consistent with the performance on the training set and achieves the expected result.

## Discussion

In this paper, we compared the performance of three classification algorithms with different input features in the signal quality evaluation of single-period pulse waves. It was discovered that a logistic regression classifier with only four input features could achieve a satisfactory result. The equation of the final prediction model reveals that a pulse wave segment will be classified as normal only when *x*_1_ and *x*_14_ are close to 0 and *x*_3_ and *x*_4_ are sufficiently large. This classifier is simple; however, it is consistent with the physiological process of the pulse wave.

**Figure 7:**
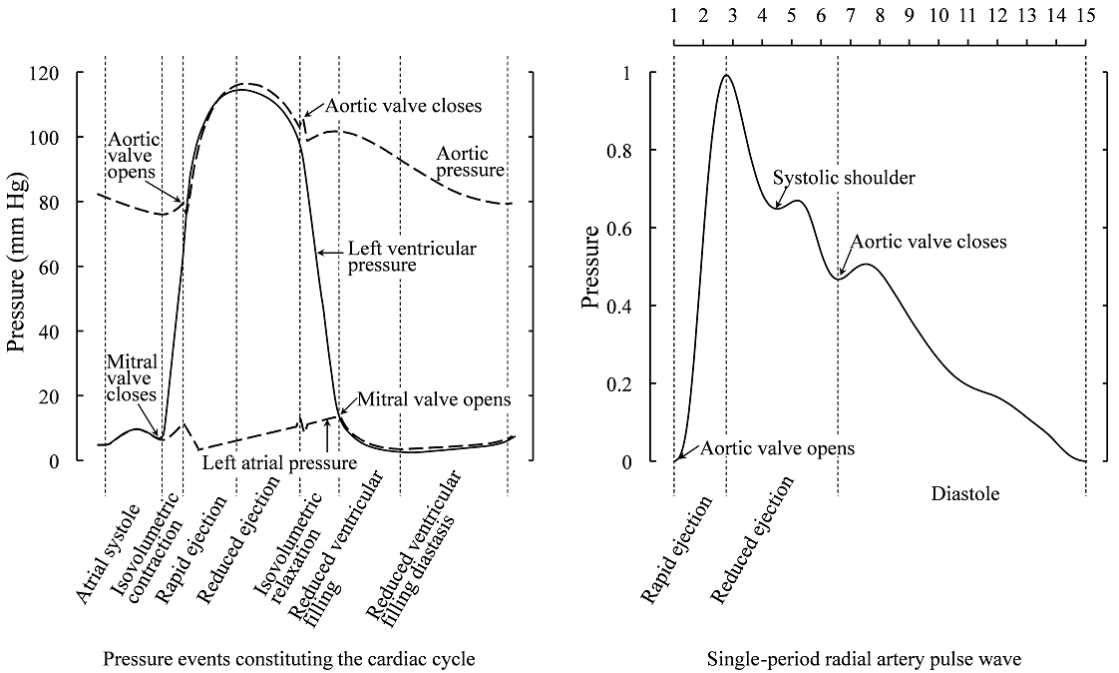
Comparison of cardiac pressure and radial artery pressure in the cardiac cycle. The fluctuation of the radial artery pulse wave corresponds to the events constituting the cardiac cycle. For a normal waveform, both the starting and ending points should be close to 0, and the peak should appear near the third feature point. mm Hg: millimeters of mercury.

A pulse wave is excited by cardiac ejection. As shown in Figure 7 [14,15], the fluctuation of the radial artery pulse wave corresponds to the events constituting the cardiac cycle. Therefore, a radial pulse wave is very similar to an aortic pulse wave. The pressure begins to rise rapidly as the aortic valve opens and ventricular ejection occurs. Shortly after ejection begins, the pressure reaches a peak and then gradually decreases. On the other side, the aortic pulse wave is greatly influenced by the reflection wave from the lower limbs, whereas the radial pulse wave is mainly influenced by the reflection wave from the upper limbs [16]. The peak of a radial pulse wave occurs much earlier than that of an aortic pulse wave due to differences in timing of the wave reflections in the upper limbs and the relatively distant lower body. When the reflection wave from the lower limbs and aortic valve closure propagates to the radial artery, the radial pulse wave correspondingly increases for a short time. These increases may not occur due to some physiological or pathological factors [17]. However, the rapid rise and gradual decline are essential features of a normal radial artery pulse wave.

For a normal waveform, it is apparent that both the start and end points should be close to 0. A high start or end point indicates that the waveform exhibits significant interference. As a result of the relatively long duration of the diastole, the pulse wave remains low and steady at the end of the diastole. Both *x*_14_ and *x*_15_ are small in normal waveforms. However, as *x*_15_ is also the starting point of the next cardiac cycle, it is more likely to increase due to cardiac activity before systole and inaccurate cycle division. *x*_14_ is thus more representative of the end point than *x*_15_. Correspondingly, at the beginning of the cardiac cycle, due to the rapid rise of the pulse wave in the systole, *x*_2_ is not more representative than *x*_1_. Therefore, *x*_1_ and *x*_14_ were incorporated into the model to indicate the conditions of the starting and ending points. In addition, *x*_1_ and *x*_14_ of an incomplete cycle will not be sufficiently small for the signal to be considered normal because the segmentation points of an incomplete cycle only contain signals of part of the cardiac cycle as opposed to both ends. Therefore, both *x*_1_ and *x*_14_ can aid identification of the qualities of segmentation.

The peak of the radial artery pulse wave usually appears near *x*_3_. Therefore, under normal conditions, *x*_3_ should be close to 1. Due to the relatively slow rate of pressure drop during systole, *x*_4_ does not have sufficient time to become very small. If either *x*_3_ or *x*_4_ is not sufficiently large, it is sufficient to prove that the waveform is abnormal. This can identify external interference; for example, the maximum value will not appear near *x*_3_ due to the elevation of the latter part of the waveform, which can also lead to an anomaly in *x*_14_. Furthermore, this change can aid the identification of segmentation errors. If we regard multiple cycles as one cycle, the first peak appears too early; as a result, *x*_3_ and *x*_4_ are not sufficiently large in most cases. If 5 or more cycles happen to be segmented together, one of *x*_3_ or *x*_4_ may be close to 1. However, under these circumstances, the waveforms rise and fall much more rapidly; thus, it is difficult for *x*_3_ and *x*_4_ to maintain large values simultaneously. Therefore, the incorporation of *x*_3_ and *x*_4_ into the model can help classify the waveform by detecting whether the peak of the waveform is located at the correct position.

In summary, the four input features of the logistic regression model are not only simple and effective but also interpretable. However, this model also has limitations. Most of our samples are free of heart disease, and the corresponding pathological characteristics will not be shown in the data. Consequently, this classifier only aimed to investigate common normal radial artery pulse waves. When searching for differences in the pulse waveforms of individuals without heart disease, we can effectively identify and eliminate abnormal segments with this classifier. However, for patients with some specific diseases, their pulse waves may change due to various pathological factors, which will ultimately lead to errors in classification. An example of this is sinus arrest, a condition in which the sinus node does not produce an impulse in one or more cardiac cycles; this causes the heartbeat to pause for a while, which will generate a long diastole segment in the pulse wave. This is a very important pathological signal; however, our model will classify it as a segmentation error due to the premature peak value. On the other hand, we may be able to use this property to improve the accuracy of some applications, such as distinguishing between premature contraction and atrial fibrillation. Premature contraction is the main cause of false positive error in AF detection algorithms [18]. There is still a certain proportion of normal segments in the pulse waves of patients with premature contraction, whereas AF will decrease the frequency of normal segments due to its irregular stroke volume and cardiac rhythm. This classifier may help distinguish the two cases by determining the ratio of normal segments to abnormal segments in the pulse wave series. In general, this classifier works well in normal cases, and its application scope can potentially expand according to its physiological significance. However, for some specific diseases, this classifier may lead to misclassification and even loss of key information. In the future, we hope to study the pulse wave characteristics of different diseases and distinguish them from random interference and the pulse wave characteristics of healthy people to subsequently improve the classifier and expand its application scope based on the new discoveries.

## Abbreviations

AF: atrial fibrillation
AUC: area under the curve
KNN: k-nearest neighbor
ROC: receiver operating characteristic
SVM-Linear: support vector machine-linear kernel
SVM-Poly: support vector machine-polynomial kernel
SVM-RBF: support vector machine-radial basis function kernel
TCM: traditional Chinese medicine
